# Adaptation of plant photosynthetic metabolism to extreme CO_2_ levels in Yellowstone revealed by *in vivo* fluorescence dynamics

**DOI:** 10.3389/fpls.2026.1774168

**Published:** 2026-07-30

**Authors:** Marie-Claire ten Veldhuis, G. Charles Dismukes

**Affiliations:** 1 Water Management Department, Faculty of Civil Engineering and Geosciences, Delft University of Technology, Delft, Netherlands; 2 Waksman Institute of Microbiology, Rutgers University, the State University of New Jersey, Piscataway, NJ, United States; 3 Department of Chemistry and Chemical Biology, Rutgers University, the State University of New Jersey, Piscataway, NJ, United States

**Keywords:** acidification, carbon dioxide, carboxylation, cyclic electron flow, environmental adaptation, plant photosynthesis

## Abstract

**Introduction:**

While CO_2_ enrichment studies have provided valuable insights into how plants respond to elevated CO_2_ levels expected under future climate conditions (up to ~700 ppm), if and how photosynthesis can adapt to more extreme CO_2_ levels in nature remains yet unknown. Here, we investigate changes in photosynthetic metabolism in a C3 plant growing near high CO_2_ emission sources in Yellowstone National Park, at levels up to 6000 ppm CO_2_.

**Methods:**

We analyze chlorophyll variable fluorescence emission kinetics to identify changes in the photosynthetic electron transport chain (PETC) between plants adapted to high CO_2_ versus control plants growing at ambient CO_2_. By applying short flashes using the Fast Repetition Rate Fluorometry (FRRF) technique we separate photochemical from non-photochemical quenching events. This enables monitoring electron transport from water oxidation within PSII into the downstream carrier pools (Plastoquinone (PQ), Ferrodoxin, and NADP^+^) of the PETC and into CO_2_ carboxylation within the Calvin Benson-Bassham (CBB) cycle.

**Results:**

We find that plants growing near the extreme CO_2_ sources in Yellowstone are much slower to overcome bottlenecks in the PETC and reach lower photochemical Fv/Fm values indicative of lower photosynthetic efficiency. We observe that these plants require higher CO_2_ levels to activate the reactions in the CBB cycle.

**Discussion:**

We conclude that plants growing near high CO_2_ sources are metabolically poised in Cyclic Electron Flow (CEF) at ambient CO_2_ concentrations and switch to Linear Electron Flow (LEF) only in high CO_2_. Second, after a brief period of dark time (3–4 minutes), the near-source plants more rapidly restore the PETC bottlenecks, slowing LEF and recovering CEF. This outcome retains electrons in the PQH_2_ pool slowing flux into the NADP^+^ pool through LEF and blocking utilization of CO_2_ by reactions in the CBB cycle. We explain this adaptive response as a coping mechanism against acidification at high CO_2_ growth conditions, which compromises enzymatic activity and proton motive force generation. We conclude that plants adapt to extreme atmospheric CO_2_ by further compromising the inefficient CBB cycle and by rerouting electron flux through the PETC from LEF into CEF to stimulate more proton pumping and ATP production.

## 1 Introduction

When atmospheric CO_2_ levels rise, plants can increase their photosynthetic productivity by suppressing the photorespiration process in which O_2_ competes with CO_2_ for carboxylation by the enzyme RuBisCO (Sharkey et al., 1988). However, long-term exposure to elevated CO_2_ is associated with other adaptations that lower stomatal opening and lower chlorophyll concentration (Dusenge et al., 2019) (Griffin and Seemann, 1996), (Sims et al., 1998). Free-air CO_2_ enrichment studies found an increase in net primary productivity and yield in C3 species, for CO_2_ levels up to ~700 ppm, under non-stress conditions (Ainsworth and Long, 2021; Ainsworth and Rogers, 2007). At even higher levels, CO_2_ can inhibit both photosynthesis and respiration by acidification, which causes accelerated plant mortality through several pathways (Maschler et al., 2022). After atmospheric CO_2_ is taken up through the cell wall, it enters the internal aqueous (aq) and lipid membrane (mem) domains as weakly associated (solvated) CO_2_, denoted CO_2_(aq) and CO_2_(mem). These forms can equilibrate rapidly across internal membranes at minimally 100 times faster rates than the anionic form HCO_3_^-^ (bicarbonate) (Gutknecht et al., 1977). The chemical reaction of CO_2_ with water (hydrolysis) followed by H^+^ ionization and additional solvation (hydration) produces aqueous bicarbonate, HCO_3_^-^(aq) and H_3_O^+^(aq). Five acids are produced in this way, including two Lewis acids (electron pair acceptors): CO_2_(aq) and CO_2_(mem), and three Bronsted acids (H^+^ donors) in the aqueous phase: H_2_CO_3_, HCO_3_^-^, H_3_O^+^ (dropping the phase notation for simplicity) (Gutknecht et al., 1977). Thus, CO_2_-induced acidification is a multi-component and multi-phase acidification phenomenon that can disrupt metabolism in multiple compartments (Ananyev et al., 2018; Maschler et al., 2022). For example, by collapsing transmembrane proton gradients, charge neutralization of membrane electrical potentials, ATP-synthase inhibition, uncoupling of ATP synthesis, and carbamylation of proteins and nucleic acids (enzyme inhibition) (Gout et al., 2001; Heber et al., 1994; Wagner et al., 1990).

**Figure 1:**
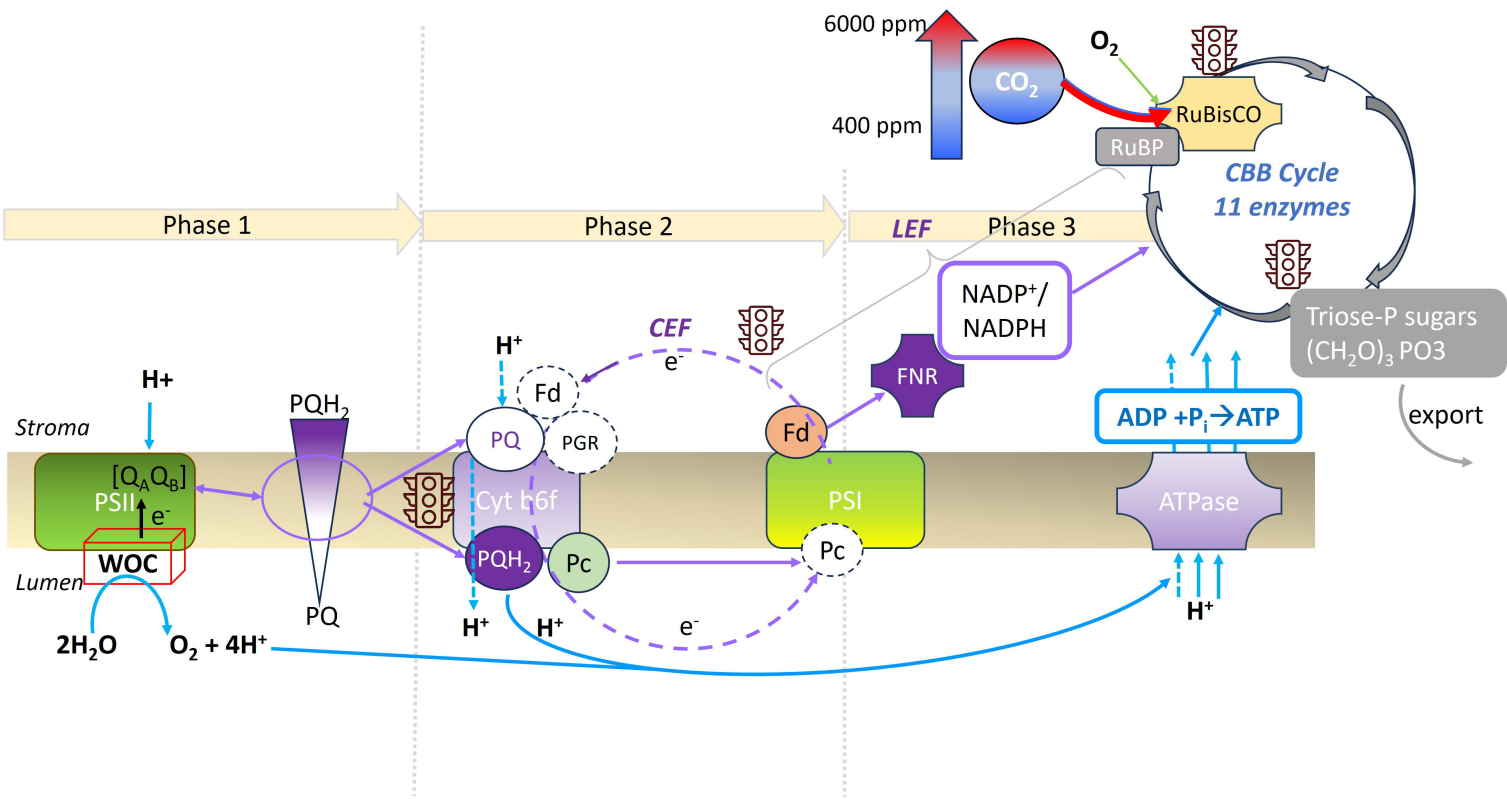
A minimalist model of the redox and proton generating steps in the photosynthetic electron transport chain (PETC) of plant chloroplasts and how they contribute to CO_2_ assimilation in the Calvin-Benson-Bassham (CBB) cycle. Purple arrows indicate linear electron flow, dashed purple arrows indicate cyclic electron flow. PQ/PQH_2_, PC, Fd, NADP^+^/NADPH and ATP/ADP are carrier pools that can temporarily store electrons and protons. “Traffic-light” symbols indicate flux bottlenecks in the PETC and CBB cycle measured by flux through PSII as follows: PQ regeneration at QB via PQH_2_ reoxidation at Cytb6f; Branching of Ferredoxin between LEF/CEF via oxidation at FNR/NADP^+^ versus oxidation at Cytb6f/PQ; Branching between CO_2_ carboxylation and O_2_ oxygenation of RuBP by RuBisCO; Branching between RuBP regeneration and export of triose phosphate from the chloroplast.

Plants sense and respond to CO_2_ levels at internal sites within the chloroplast, mitochondrion, and cytoplasm. In the chloroplast, there are four forms of dissolved aqueous CO_2_: [CO_2_]_aq_, H_2_CO_3_, HCO_3_^-^, and CO_3_^2-^ that can regulate and modify the fluxes of electrons and protons at numerous sites. This includes the photosynthetic electron transport chain (PETC), which regenerates the principal reductant and energy carrier molecules, NADPH and ATP, consumed during cellular biosynthesis and repair (Ananyev et al., 2018; Kramer and Evans, 2011). Dissolved CO_2_ also modifies the enzymes and intermediates of the Calvin-Benson-Bassham (CBB) Cycle, which is comprised of 11 enzymatic reactions, including ribulose-bisphosphate carboxylase-oxygenase (RuBisCO), that perform the carboxylation/oxygenation reactions of 1,5-ribulose bisphosphate (RuBP) and its regeneration from triose phosphate molecules (**Figure 1**). A comprehensive understanding of the PETC has emerged that describes the photoreactions starting from water oxidation and resulting in photophosphorylation (ATP production) and NADP^+^ reduction, which in turn power the carboxylation reaction and the CBB cycle (Prywes et al., 2023; Raines et al., 2022; Sharkey, 2024) (**Figure 1**). Three rate-limiting processes (chokepoints) have been identified in relation to CO_2_ carboxylation (Kiirats et al., 2009): First, the availability and relative generation rates of ATP and NADPH can become rate-limiting at low light intensity as they provide the required energy and reductant in the CBB cycle. This is also referred to as the light-limited electron transport rate (Farquhar et al., 2001). Second, the flux of CO_2_ conversion through carboxylation depends on at least four concentrations: the activated fraction of RuBisCO, the available concentrations of CO_2_ and RuBP (the CO_2_ receiver molecule) which binds to the carboxylating enzyme RuBisCO, and the available O_2_ concentration. Because CO_2_ and O_2_ compete for reduction by RuBisCO (the ratio of fluxes ~100:1 (Prywes et al., 2023), the carboxylation rate can change with light intensity owing to O_2_ evolution by PSII (Dusenge et al., 2020; Qian et al., 2021; Wu et al., 2023). Third, at saturating CO_2_ and high light intensities, the regeneration rate of RuBP in the CBB cycle limits the rate of carboxylation, owing to the export of the triose phosphate product out of the chloroplast (Schreiber et al., 1995; Sharkey, 2024; Suzuki et al., 2020). In theory, the highest efficiency of light and CO_2_ utilizations can be achieved when these three processes are kinetically in balance, i.e. synchronized to avoid reverse reactions and build-up of intermediates that are targets for competing reactions including the formation of harmful reactive oxygen species (ROS) (Kramer and Evans, 2011; Sweetlove and George Ratcliffe, 2011). This kinetic balance is so rarely encountered *in vivo* that it is a main contributor to the low overall solar energy utilization efficiency of photosynthesis (Blankenship et al., 2011). This reinforces the need to understand how short-term acclimation and long-term adaptation to elevated CO_2_ influence the kinetic and energetic efficiencies of these reactions.

We investigate adaptation to elevated environmental CO_2_ levels in a C3 wild plant (*Prunella vulgaris*) growing next to natural springs that release high amounts of CO_2_ (up to 6000 ppm) into the atmosphere at two sites in Yellowstone National Park (United States). The sites were selected as stable gaseous CO_2_ sources and absence of other acidic gases according to chemical assays over at least the last two decades (Bergfeld et al., 2014; Hurwitz et al., 2020; Werner and Brantley, 2003): Mammoth Terraces date back to several hundred thousand years, the Gibbon Geyser Basin dates to 400,000 years ago (Bargar, 1978). These areas have been generating travertine since the retreat of glaciers in the last ice age, circa 15,000 years ago (Bargar, 1978). We examine changes in a range of photosynthetic variables measured on leaves in the field to investigate the effect of external CO_2_ levels on PETC reactions. Additionally, we apply Fast Repetition Rate Fluorometry on leaf samples to measure the degree of obstruction occurring at multiple chokepoints in the PETC revealed by dynamic changes in chlorophyll-a (Chl-a) variable fluorescence in response to a customized flash protocol.

We aim to answer the following question: how does photosynthetic metabolism adapt upon exposure to extreme CO_2_ growth conditions? We collect physiological observations and study the kinetics of the PETC to examine three current hypotheses that have been phrased in the literature for long-term adaptation to high environmental CO_2_: 1) whether adaptation to higher CO_2_ enhances the carboxylation rate in the CBB? 2) whether the maximum carboxylation rate associated with the functional RuBisCO content is lowered? 3) whether plants can adapt to the elevated transport of CO_2_ across cell walls and membranes that cause potential harm due to acidification?

## 2 Methods

### 2.1 Sampling sites in Yellowstone National Park

**Figure 2:**
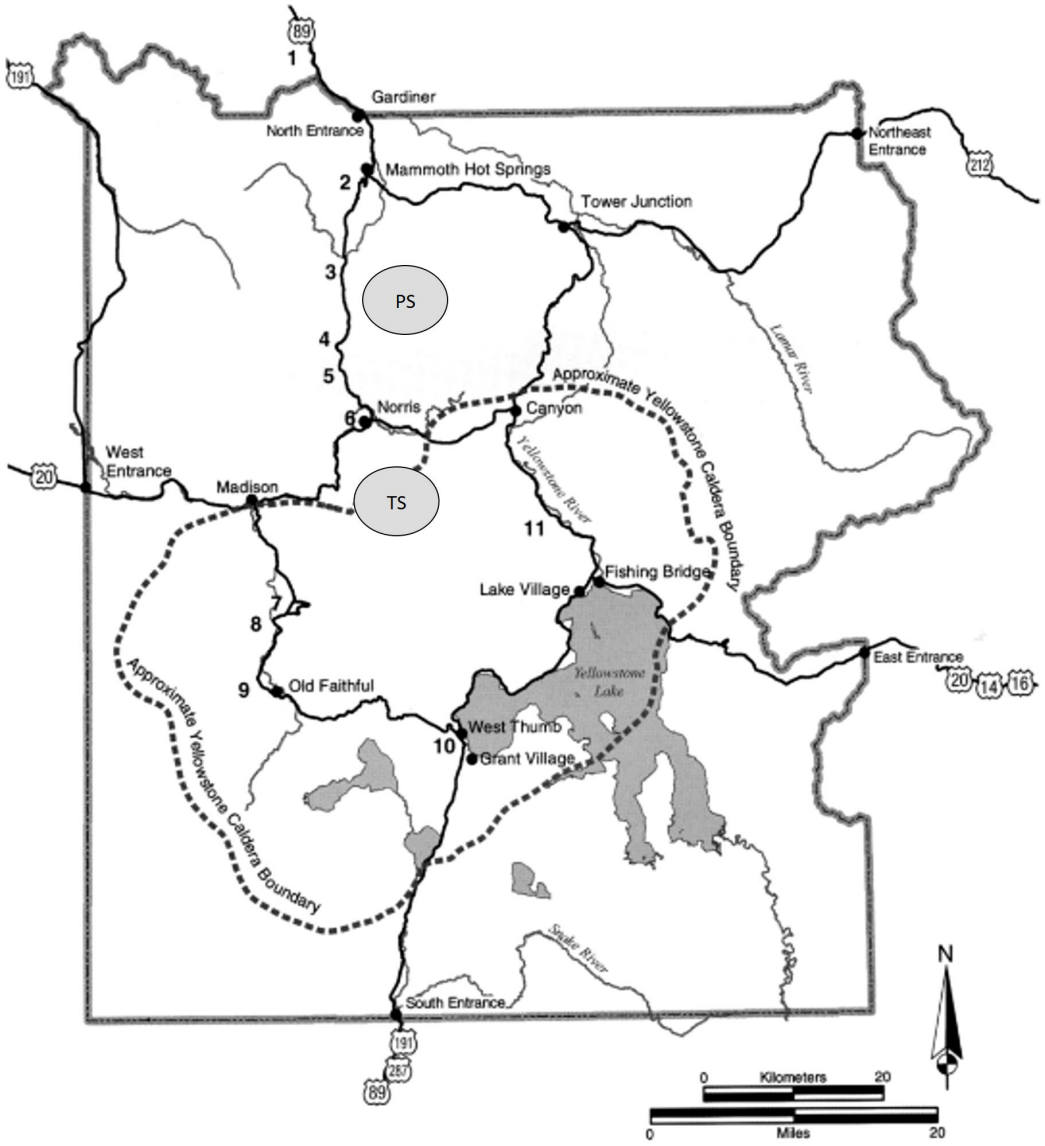
Sampling sites at Yellowstone National Park: Terrace Springs (TS) and Poison Spring (PS) at Mammoth Terrace. Source: National Park Service, Yellowstone National Park.

*In-situ* measurements were conducted and whole plant samples collected (*Prunella vulgaris*) at two sites in Yellowstone National Park (YNP), Terrace Spring (TP) in the Gibbon Geyser Basin, and Poison Spring at Mammoth Terrace (**Figure 2**). At these sites, the gas composition at the vent sources is very high in CO_2_ (96.1-99.8% normalized dry gas composition), while low in water vapor (10.3-37.7%), combined with low levels of CH_4_ (0.0002% and 0.002% at TP and PS), He (0.003%) and H_2_S (<0.01% TP, 0.03% PS) (Bergfeld et al., 2014; Hurwitz et al., 2020; Werner and Brantley, 2003). Measurements were conducted close to the high CO_2_ sources (~6000 ppm CO_2_ at low wind conditions) up to a distance of about 10 meters from the point source where CO_2_ concentrations were down to ambient (~400 ppm CO_2_). Soil and air temperatures varied between 22 and 26 °C on the days of sampling. Sampling sites were well-drained (located above the spring water surface). Our sampling sites were sufficiently close (~10 m distance) to expect soil nutrient conditions to be the same for all plants sampled herein.

### 2.2 Plant materials

*Prunella vulgaris* (Young-Mathews, 2012) is a perennial, low-growing, flowering C3 plant that is native to North America and is found throughout YNP in diverse environments. It is one of the few species that is found within one meter of elevated CO_2_ sources as well as in control conditions at ambient CO_2_. For each specimen collected in the field, we measured CO_2_ concentration in the atmosphere surrounding the plants, ground temperature at the sampling sites, and leaf stomatal conductance (SC-1 leaf porometer; Meter Group in 2022, LI600 porometer LICOR in 2024). Atmospheric CO_2_ was consistently above 6000 ppm at the CO_2_ source. CO_2_ levels fell to ambient (~400 ppm) over a distance of 10 meters from the source, in wind-still conditions. When the wind picked up later in the day, CO_2_ levels fluctuated due to atmospheric mixing with CO_2_ levels between 1500 ppm to above 6000 ppm in the area around the source. We restricted our field measurements (handheld device, Section 2.3, **Table 1**) to wind-still conditions. Soil temperatures at field sites varied between 20 to 25°C. The stomatal conductance values measured in the field ranged from 0.7 to 1.0 mol m^-2^ s^-1^.

**Table 1:** Summary data collection for *in-situ* observations (MultispeQ) and lab measurements (FRRF protocol).

Instrumentation	Data collection year 2022	Data collection year 2024
MultispeQ instrument, *in-situ* leaf measurements at field site	–	Four distances relative to CO_2_ source - < 10 cm: 8 - < 100 cm: 25 - < 250 cm: 16 - ~ 10 meters (Control): 28
FRRF protocol, lab measurements, on leaf samples from plants collected at field site	24 whole plant samples, 14 accepted for analysis Leaf samples of plants at two distances relative to CO_2_ source: - < 100 cm: 18 - ~ 10 meters (Control): 13	20 whole plant samples, 16 accepted for analysis Leaf samples of plants at two distances relative to CO_2_ source: - < 100 cm: 18 - ~ 10 meters (Control): 27

Intact plants with roots were collected during two field visits, between 13 and 18 July 2022 (24 plants) and between 10 and 20 July 2024 (20 plants) (**Table 1**). Sampling was conducted under the supervision of the NPS (Permit nr.: YELL-2021-SCI-8098). Plants that were damaged during harvesting or transport were discarded, a total of 14 (2022) and 16 (2024) plants were accepted for further examination within 2–6 hours from collection at the field sites.

### 2.3 Instrumentation and measurement protocols

MultispeQ: this portable device manufactured by PhotosynQ combines several tools into one handheld device for leaf-based measurements in the field. In this study we applied the RIDES 2.0 protocol that measures PSII-related variables based on variable chlorophyll-a fluorescence using a PAM-type fluorometer, a chlorophyll meter estimates chlorophyll content based on SPAD-values (a measurement that reflects the relative greenness of a leaf, which correlates with its chlorophyll-a concentration) and a spectrometer that measures PSI-related variables. The protocol also measures a set of physiological variables, including leaf thickness and leaf temperature as well ambient variables including incoming radiation, air temperature and humidity. Details on the MultispeQ device and RIDES protocol are provided in (Kuhlgert et al., 2016).

FRRF: we used the Fast Repetition Rate Fluorometry (FRRF) technique to monitor kinetic phases of fluorescence induction in relation to CO_2_ exposure. The variable yield of Chl-a fluorescence emission (Fv) reflects primarily changes in the redox state of Q_A_, the primary quinone electron acceptor of PSII (Duysens and Strehler, 1963). The Fv yield further depends on prior illumination history (NPQ (Gorbunov et al., 2011), and the conformational state of PSII (Schansker et al., 2014), temperature, and the redox state of Q_B_ (Gorbunov et al., 1999). All factors are fixed or eliminated in the FRRF protocol that we have adopted, the details of the method are fully described in (Ananyev et al., 2026).

The FRR flash protocol applied in this study consists of trains of 50 flashes (80 μs flash duration) applied at 100 Hz flash frequency to produce 50 photochemistry-induced variable fluorescence (pFv) recordings separated by 10 ms inter-flash time. This flash time interval is much longer than any of the donor side reactions from water oxidation which ensures these reactions have reached their end states (S-states). The photon flux density during a single flash is constant at 2000 μmol m^-2^ s^-1^. The variable fluorescence yield per flash (pFv/pFm) is a measure of the oxidation state of Q_A_/Q_A_^-^, the degree of openness of PSII. We use pFv for Fv derived from Fo and Fm per flash in FRR fluorometry to clarify that this refers strictly to photochemically induced variable fluorescence. By computing the ratio pFv/Fm per flash in FRR, the effect of NPQ is canceled out, leaving only the transient *photochemical* part that contributes during the 80 µs flash (as explained in detail in (Ananyev, in press)).

**Figure 3:**
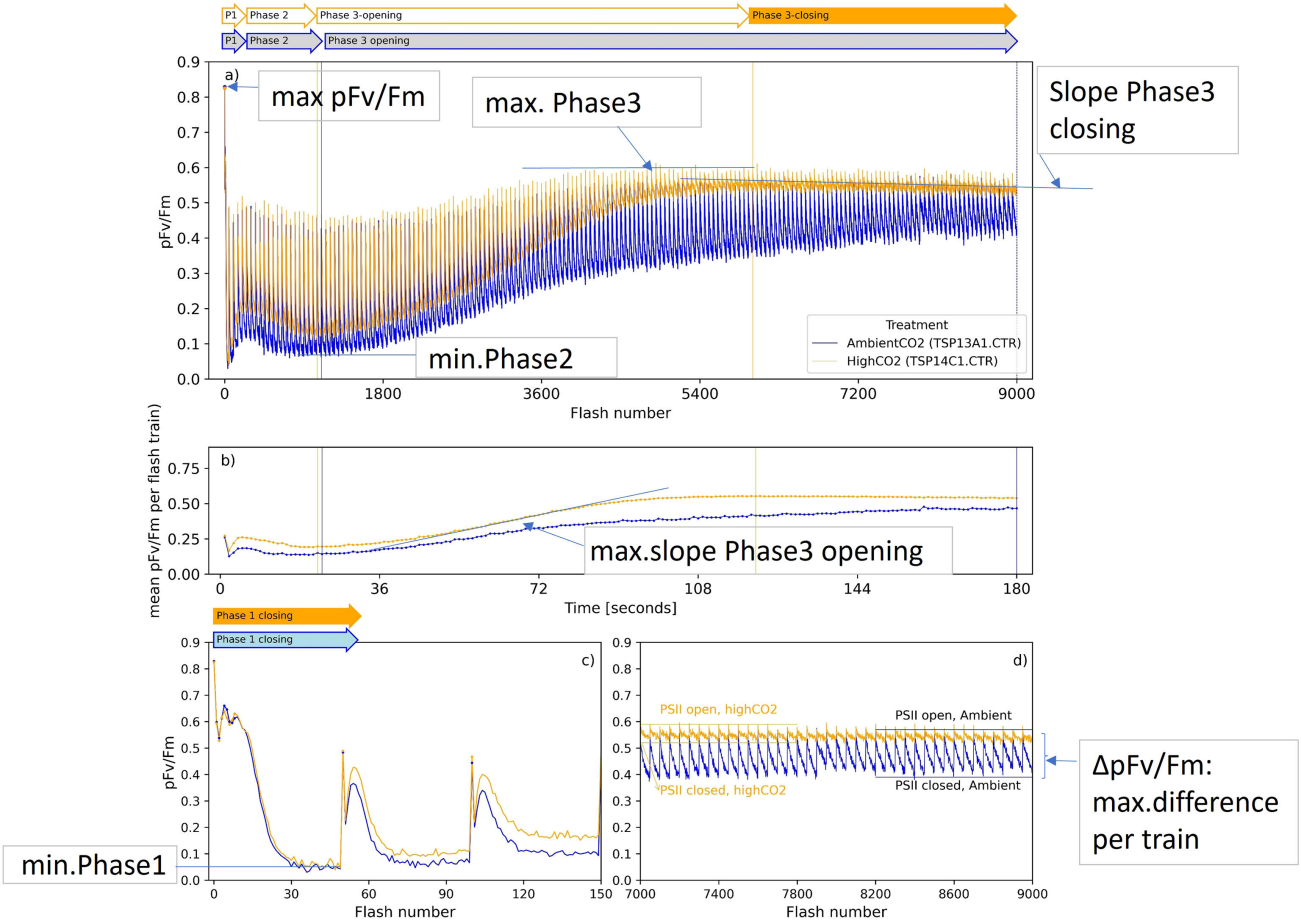
*Prunella vulgaris*: illustration of *photochemical* Fv/Fm (pFv/Fm) data collected by the FRRF protocol for 2 sample leaves, one exposed to ambient CO_2_ (400 ppm) and a second leaf exposed to high CO_2_ (6000 ppm). Showing **(a)** all 9000 flashes (180 flash trains); **(b)** integrated pFv/Fm per flash train; **(c)** first three flash trains; **(d)** last 2000 flashes (40 flash trains). Annotations indicate quantitative values derived from the protocol: max pFv/Fm, pFv/Fm-value for first flash; Min.Phase 1 representing maximum filling of the PQ/PQH_2_-pool, min.Phase 2 representing filling of the second pool, Fd/Fd^-^ reduction state; max.slope Phase3, maximum increase in pFv/Fm during Phase 3 opening, representing the rate of electron transport in the PETC in relation to the CO_2_ dependent reaction rate in the CBB-Cycle; Max.Phase3 associated with max electron flux through the CBB at the given light intensity and CO_2_ concentration, Slope Phase3 closing, slope of mean pFv/Fm trace over the last 50 trains of Phase 3, representing decrease in electron transport rate due to downstream limitations, ΔpFv/Fm, difference between maximum and minimum pFv/Fm per flash train.

The flash train protocol is illustrated in **Figure 3**. The evolution of pFv/pFm over the course of the first train of 50 flashes measures the degree of availability of electron acceptors for Q_A_^-^ (Q_B_ and PQ-pool), from the dark-adapted population on the first flash (pFv/Fm ~0.85) down to the 50^th^ flash (pFv/Fm ~0.05). Period-4 oscillations in pFv/Fm for the first 4–10 flashes reflect oscillations in the S-States in the Water Oxidation Complex, the electron donor to P680^+^ (Ananyev and Dismukes, 2005), **Figure 3c**). The second and onward flash trains probe slower reactions that can modify the flux of electrons out of PSII, notably flux out of Q_A_/Q_A_^-^ to downstream pools of carriers in the PETC and the CBB-Cycle. The full protocol consists of 180 trains of 50 flashes each, separated by 0.5 second dark time. This corresponds to an average incident photon flux density of 8 μmol m^-2^ s^-1^ throughout the 3-minute duration of the protocol. The train interval time is selected to measure kinetics of downstream reactions of the PETC that allow or suppress reoxidation of Q_A_^-^. This protocol enables monitoring both fast and slow changes of fluorescence induction, from initial oscillations in pFv and Fm from the S-States in the PSII-WOC to the slower downstream reactions of the PETC. To investigate the recovery of the fluorescence induction kinetics in the dark, we ran two experiments of 180 trains each in sequence on the same sample, separated by an intermediate dark time of 4 minutes.

### 2.4 Data processing and statistical analysis

MultispeQ data were collected in the field using the device’s RIDES 2.0 protocol on attached leaves of *Prunella vulgaris* plants growing near high CO_2_ sources at distances between 10, 100 and 250 cm from the source, as well as under control conditions (~400 ppm CO_2_). Variables that were derived from the RIDES 2.0 protocol observations include leaf thickness, SPAD values, Phi(II) and Phi-NPQ, PSI open and oxidized centers.

FRRF data were collected on whole plants after transport to the lab within 2 to 6 hours. Fresh leaves were removed in the lab for each sample and mounted to the sample chamber of the FRRF instrument. Features that were derived from the FRRF data are summarized in **Table 2**. They include features (maxima, minima and slopes) that report on three kinetic phases of the PETC (**Figures 1**, **3**): Phase 1 associated with electron transport from the Water Oxidation Complex into PSII and the PQ-pool; Phase 2 reflecting the kinetics of electron transport between the PQ-pool and PSI via Cytb6f; and Phase 3 representing the transport of electrons to and from NADP^+^/NADPH.

**Table 2:** Features extracted from FRR Fluorescence emission data for kinetic phases of the variable fluorescence traces.

FRRF extracted feature	Feature description
Max pFv/Fm	First pFv/Fm value for Train 1. For fully dark-adapted samples, this represents the maximum quantum yield of PSII, ΦII_max_.
Min Phase 1	The first minimum pFv/Fm value, representing maximum filling of the PQ/PQH_2_-pool (**Figure 1**), generally reached within Train 1.
Min Phase 2	The second minimum pFv/Fm value, representing filling of the second pool, Fd/Fd^-^ (**Figure 1**).
Max slope Phase 3 opening	Maximum increase in pFv/Fm during Phase 3 opening, representing the rate of electron transport in the PETC in relation to the CO_2_ dependent reaction rate in the CBB-Cycle
Max Phase 3	Maximum pFv/Fm reached in Phase 3, representing the maximum electron transport rate under the given experiment conditions (especially CO_2_ exposure level)
ΔpFv/Fm per train	Difference between maximum and minimum pFv/Fm per flash train. This difference is a measure of the rate of oxidation of the PQH_2_ pool by downstream reactions.
Slope P3-closing	Slope of mean pFv/Fm trace over the last 50 trains of Phase 3, representing decrease in electron transport rate due to downstream limitations, esp. TPU-limitation.

Statistical tests that were performed on the datasets to identify differences between control plants and plants growing near the CO_2_ sources. First, a Kruskal-Wallis test for significant differences between datasets, followed by a Mann-Whitney U test for pair-wise comparison of datasets. Both tests are non-parametric, avoiding assumptions about normality of the datasets. All tests were conducted at the 5% (p ≤ 0.05) significance level.

## 3 Results

### 3.1 Leaf measurements of photosynthesis variables in the field

**Figure 4:**
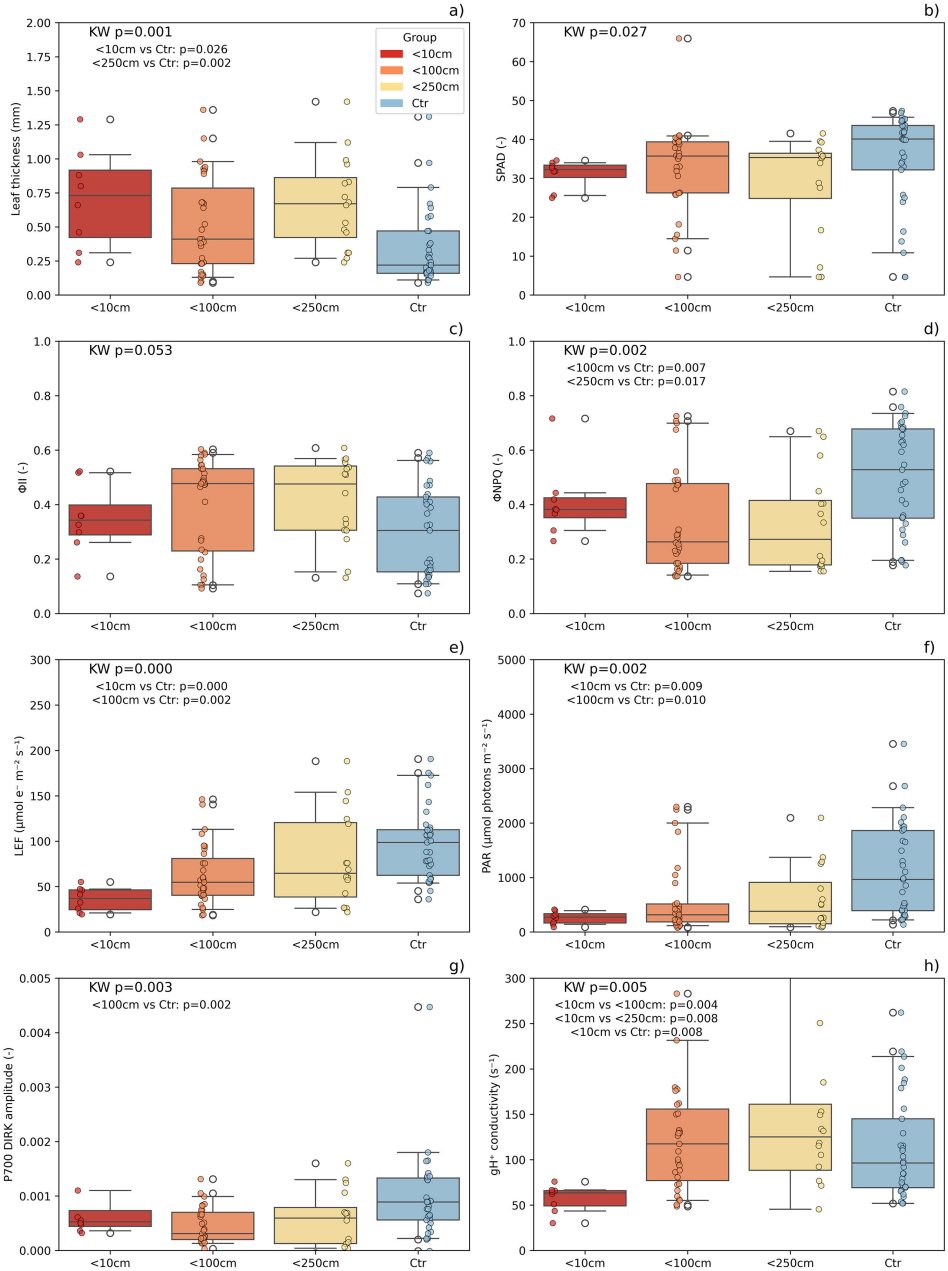
*Prunella vulgaris*: boxplots of leaf thickness and SPAD values **(a, b)**, Phi2 and NPQ **(b, c)**, LEF and PAR light intensity **(e, f)**, oxidized P700 and proton conductivity gH+ **(g, h)** for control plants and for plants growing within 10, 100 and 250 cm of the high CO_2_ source. Boxes span the 25-75%-ile range, whiskers extend to the 5-95%-ile range. Significant differences were tested based on a global Kruskal-Wallis and pair-wise Mann-Whitney U tests.

First, we compare leaf thickness and SPAD values of *Prunella vulgaris* leaves, measured on leaves of plants at three distances along a gradient in CO_2_ from the emission source and at further distance of control plants growing at ambient, ~400 ppm CO_2_ (**Figures 4a, b**). Leaf thickness was higher for plants growing near the high CO_2_ source at the 5% significance level for plants within 10 and 250 cm from the source, compared to control plants, though a large spread in thickness values was recorded in each of the groups. Leaf SPAD values were not significantly different between the four groups of plants. Phi(II) maximum (Phi2) values range between 0.1 and 0.6, and PhiNPQ values between 0.1 and 0.85. Phi(II) values were not significantly different between sample groups, while NPQ was lower in the group of plants growing within 100 and 250 cm from the CO_2_ source compared to the Control group (**Figures 4c, d**). Both variables show a large spread, likely associated with the wide range of PAR conditions that the leaves were exposed to in the field (**Figure 4f**). Linear Electron Flow (LEF) is calculated from Phi(II) and PAR combined in the PAM-based protocol, under the assumption of a fixed % absorption of incident light and equal energy distribution between PSI and PSII. Mean LEF is increasing systematically with distance from the CO_2_ source, up to 3-fold compared to the control (**Figure 4e**). This suggests that plants in the high CO_2_ growth environment achieve lower electron flux from water to NADP^+^, or alternatively distribute absorbed light differently between PSI and PSII (violating the underlying assumption for the LEF calculation). **Figure 4g** reveals a significant difference for the amount of oxidized PSI (P700) only for the <100cm versus the control group. Membrane proton conductivity (gH^+^), is significantly lower for the plants growing within 10cm of the source (gH+), compared to all other groups. Lower values can be an indication of bottlenecks in the PETC, lower electron flux through PSI and proton flux through ATP synthase, respectively.

### 3.2 Changes in PETC kinetics in plants at extreme CO_2_ growth sites

**Figure 5:**
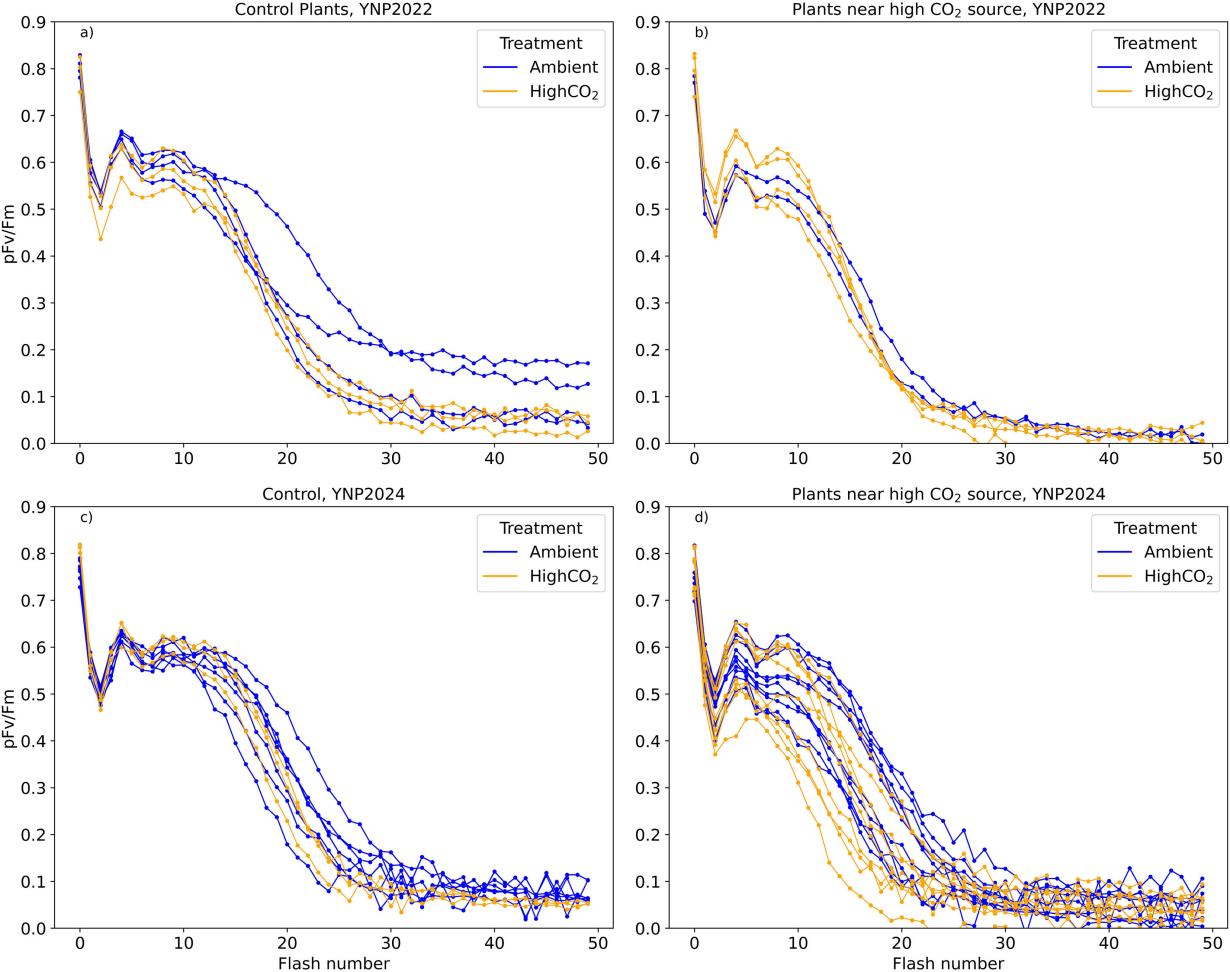
*Prunella v*. leaf samples: pFv/Fm data collected by the FRRF protocol for Control plants **(a, c)** and plants growing near high CO_2_ sources **(b, d)**, collected during field visits in 2022 **(a, b)** and 2024 **(c, d)**. pFv/Fm values are shown for the first flash train after 60 minutes dark adaptation. Leaves were measured in ambient (400 ppm, blue traces) and in high CO_2_ (6000 ppm, orange traces).

Next, we analyze the *photochemical* Fv/Fm kinetics (pFv) from *Prunella v.* plants, measured using the FRR protocol as described in section 2.3 and illustrated in **Figure 3**. We present results for the first flash train in **Figure 5**, for leaves from control plants and from plants growing within 100 cm of a high CO_2_ source.

After dark adapting to a stationary state (60 min), pFv/Fm values decrease from the maximum initial value of ~0.83 to a local minimum on the first three flashes, then shows an oscillating pattern that continues over the first 10 or more flashes with flash period of 4. After this pFv/Fm gradually falls to a minimum level between 0.05-0.1 within ~30 flashes, up to 0.2 for two of the Control traces. This decline reflects the gradual closing of PSII that is attributed to filling of the PQ-pool (**Figure 3**, and (Ananyev et al., 2026). The initial oscillating pattern with period 4 is due to the S-state dependence of the flux of electrons and protons from the Water Oxidation Complex into Q_A_ (Ananyev and Dismukes, 2005). The maximum PSII quantum yield is 0.8 for the Control samples in both ambient and high CO_2_ (median values across samples) and 0.77 and 0.78 for the near-source samples, for ambient and high CO_2_ treatments, respectively. Note the significantly higher values compared to the Phi(II) maximum values measured in the field on leaves under light-exposed conditions. The pFv/Fm values at the beginning of the train (flashes 1-3) and the subsequent period-4 oscillations are identical for the Adapted and Control plants and independent of high versus ambient CO_2_ treatments. This means that the yield of water oxidation that forms the first three microstates: Q_A_Q^-B^, Q_A_Q_B_^2-^, Q^-A^Q_B_^2-^) and their subsequent oxidation by the PQ pool is invariant across these samples.

**Figure 6:**
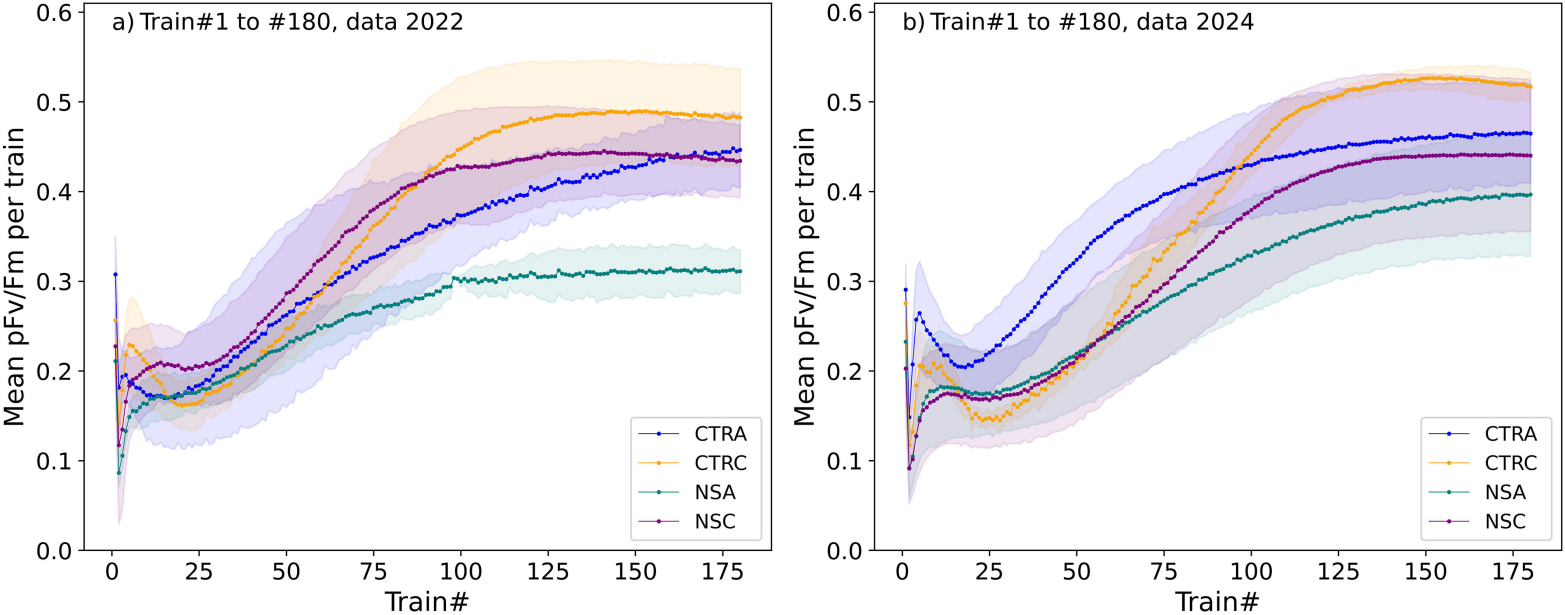
As in **Figure 5**. Traces show mean and standard deviation for each set of experiment treatments) calculated for mean values per flash train (50 flashes at 100 Hz), for 180 trains applied in sequence with 0.5 in between trains (total experiment time 3 minutes). CTRA, Control samples in ambient, CTRC, Control in High CO_2_, NSA, near-source samples in ambient, NSC, near-source samples in high CO_2_. Data collected during field visits in YNP in 2022 **(a)** and in 2024 **(b)**.

**Figure 6** shows the mean pFv/Fm per train (average of 50 flashes) for the first 180 trains using the flash protocol explained in section 3.3. This lower time resolution format focuses on the slower phases in the PETC. After the first minimum, pFv/Fm first rises to a local maximum then falls to a second local minimum around 0.15-0.2 after 15–25 flash trains. The time it takes to reach this second minimum is a measure of the time it takes (measured by the number of flash trains) to transfer electrons from the reduced PQH_2_ pool to the next photochemical acceptor (FD via PSI). It varies considerably between sample groups and treatments. For control plants, it takes 15 trains to reach the 2^nd^ local minimum for samples in ambient CO_2_, which delays to 20 trains for samples in high CO_2_ (blue and orange traces, respectively). Traces for plants growing near the extreme CO_2_ source, exhibit only a weak maximum and shallow second minimum that occurs between 20–25 trains (grey and pink traces).

After the second minimum, pFv/Fm levels rise until they reach a plateau value, or nearly so before ending the experiment at 180 trains. In ambient CO_2_ the rising slope is much faster for Control leaves (~0.004 pFv/Fm units per second, median across samples) compared to the near-source samples (~0.002 units s^-1^). Upon exposure to high CO_2_, the slope increases significantly for near-source leaves (to ~0.004 units s^-1^). The near-source samples recover similar slope and plateau values compared to the Control when exposed to high CO_2_. The increase in the rising slope upon exposure to high CO2 suggests this kinetic is associated with the removal of electrons from the PETC by the reduction of CO_2_ carboxylation reactions in the CBB-Cycle, as further confirmed in (Ananyev et al., 2026).

The maximum pFv/Fm values reached at the plateau or the end of the 180 trains vary between 0.3-0.4 for the near-source leaves in ambient CO_2_ and between 0.4 and 0.5 for the other groups. This means that after reaching the steady-state at the 8 µmol m^-2^s^-1^ light intensity of these experiments, the closure of PSII is 40-50% of the maximally open (dark-adapted) pFv/Fm value at the start of the experiment. For the other sample groups, 55-65% of the initial pFv/Fm is recovered, indicating an 15% average loss in electron flux out of the PETC to the CBB/CO_2_ receiver.

We note that for the high CO_2_-exposed samples, a weak negative slope appears over the last ~30 trains of the experiment, suggesting a downstream kinetic bottleneck in the PETC or the CBB that closes PSII.

**Figure 7:**
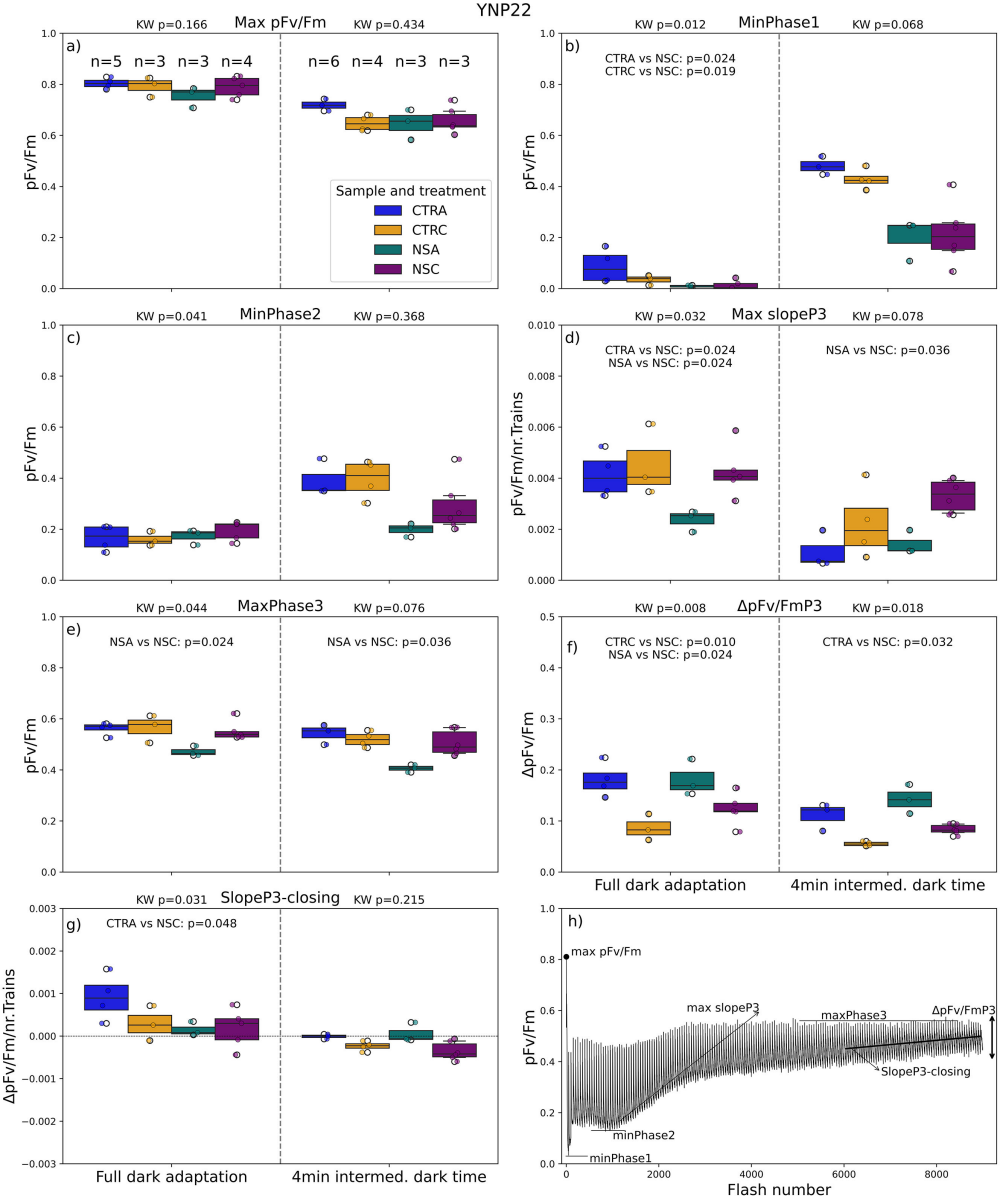
*Prunella vulgaris*: Summary of features extracted from pFv/Fm traces after two different pre-incubation dark times: 60-minute (left columns) and 4-minute (right columns). Results for leaves of Control (CTR) and Near-Source plants (NS) measured in ambient (CTRA, NSA) and high CO_2_ (CTRC, NSC). The definition of the features is illustrated in subpanel h) and explained in detail in Section 2.3. Boxes span the 25–75%-ile range, whiskers extend to the 5–95%-ile range. Significant differences were tested based on a global Kruskal-Wallis and pair-wise Mann-Whitney U tests.

A kinetic feature that shows strong sensitivity to CO_2_-exposure is the difference in pFv/Fm-values between the start and end of each flash train. As illustrated in **Figure 3**, this value decreases until reaching its smallest value at the end of the 180 trains. This pFv/Fm difference per train reflects a kinetic balance between delivery of electrons into the PETC from PSII, versus the removal of electrons from NADPH and FD by reducing CO_2_ in the CBB-cycle. The pFv/Fm difference decreases from 0.75-0.8 for the first flash train to 0.15-0.2 at the end of the 180 trains for leaves in ambient CO_2_, while for high CO_2_-exposed samples it falls to 0.08 (Control) and 0.12 (near-source). The statistics of these and all other kinetic features discussed herein are presented in **Figures 7a-f** in the form of boxplots per sample group. Boxplots in the left column show results for leaves that were dark adapted for 60 minutes prior to the experiment and for which the traces are shown in **Figure 6**.

Next, we applied two flash trains in sequence, separated by a 4-minute dark time in order to investigate slower reoxidation kinetics at this 4-minute time-scale. Results are presented in **Figure 7**, in the right-hand column of each of the panels (**Figures 7a–f**). Clear differences appear in particular for the minima in Phases 1 and 2, the rising slope for Phase 3 and the slope at the end of Phase 3 (**Figures 7b, c, e, h**). Control samples show higher minimum pFv/Fm in Phases 1 and 2, indicating that Q_A_^-^ remains more oxidized, thus PSII remains more open. Rising slopes in Phase 3 are less steep for control samples while they change little for the near-source plants. All samples except near-source plants in ambient CO_2_ conditions show a less positive or even a negative slope at the end of Phase 3, indicating presence of a downstream bottleneck for electron transport in the PETC.

In summary, two distinct differences between control plants and plants growing near the high CO_2_ source appear from the boxplots. First, leaves from near-source plants, when exposed to ambient CO_2_ reach lower pFv/Fm values in Phase 3, have a slower rising slope and a weaker slope at the end of the 180 trains, compared to control leaves. These differences point towards an adaptation or acclimation to the high CO_2_ growth environment. When exposed to high CO_2_, these samples recover similar pFv/Fm kinetics compared to the control samples. Second, after short-term (4-minutes) dark adaptation, Control samples show higher minima in Phase 2, indicating that this bottleneck remains more open compared to the fully dark-adapted state. This does not occur for the near-source plant leaves, where the minimum pFv/Fm is as low as after full dark adaptation. We infer from the higher Phase 2 minima in Control leaves after pre-illumination, that the electron flux out of the reduced PQH_2_-pool is faster compared to full dark adaptation. The acceleration is consistent with retention of Linear Electron Flow in Control leaves coming out of the light-adapted state (Belyaeva et al., 2019). The 4-minute dark time is short enough that LEF remains active in the Control plants, however this does not occur in the near-source plants, suggesting that LEF deactivates more rapidly or reverts to CEF more rapidly in the dark. By contrast, both near-source and control groups behave similarly when exposed to CO_2_, exhibiting a CO_2_-dependent acceleration of Phase 3 opening and Phase 3 closing. In the Discussion, we relate these observations to the standard model of the PETC and CBB cycle in **Figure 1**.

## 4 Discussion

The conclusions drawn from these experiments benefit from two instruments with complementary capabilities; a rugged field instrument designed for rapid multiple spectroscopic assays at low-precision (MultispeQ), and a precision benchtop fluorometer dedicated to microsecond timescale Chl-emission under controlled conditions (FRRF). Both sets of results show that a photosynthetic angiosperm (*Prunella v.*) growing near high CO_2_ emission sources (6000 ppm) acclimates to the high CO_2_ environment in several ways.

*Interpretation of the field experiments with MultispeQ.* Of the six measured variables using the MultispeQ, the derived quantity called LEF exhibits the largest change, a 3-fold decrease in mean value for plants closest to the CO_2_ source. Although the variance increases with distance, the change in mean value is statistically significant, hence firm conclusions can be drawn. The LEF quantity, as defined in the instrument protocol, is the flux passing through Linear Electron Flow (Kuhlgert et al., 2016). The 3-fold decrease in LEF together with the absence of change in quantum yield of PSII charge separation on the first flash (proportional to the variable PhiII) are interpreted as follows. Normal primary charge separation occurs, but secondary charge separation is 3-fold reduced, due to LEF. This outcome is consistent with an increase in CEF derived from the FRRF observations described further below. The quantity proton conductance gH^+^ decreases by two-fold for plants close to the CO_2_ source relative to those at distance, while the variance increases 3-fold. We choose not to draw firm conclusions from this variable, although we note that lower conductance suggests a collapse of the thylakoid pH gradient.

*Interpretation of the kinetics measured with FRRF.* Given the uncertainty in NPQ, gH^+^ and LEF observations in the field, further experiments were conducted using the FRRF to shed light on the potential role of bottlenecks in the PETC. FRRF experiments using an 80 µs pulse to sample the photochemical contribution to pFv from leaf samples identified Phase 2 and 3 bottlenecks as responding to the high CO_2_ growth environment. This results in a strong decrease in photosynthetic efficiency in leaf samples of the near-source plants when exposed to ambient CO_2_ (400 ppm, a 15-fold decrease compared to 6000 ppm in their growth environment). The decrease is associated with the Phase 2 bottleneck branching point between LEF towards NADP^+^ reduction and CEF toward PQ reduction. At the same time, electron flux from water oxidation to PSII measured through period-4 oscillations in pFv/Fm is not affected, confirming a sink limitation is the source of reduced photosynthetic efficiency, not the input from PSII.

Additionally, our observations suggest that LEF gets more rapidly deactivated in the dark in near-source plants compared to the control plants. In general, environmental factors that limit the development of sink strength predispose plants to a greater acclimation of photosynthetic capacity, and lessen the stimulation of photosynthesis by growth at elevated CO_2_ ( (Leakey et al., 2009), and references therein). Field studies have since confirmed that reduced or insufficient sink capacity from environmental, genetic or management practices leads to an increase in foliar carbohydrates, and subsequent down-regulation of photosynthetic capacity (Ainsworth and Long, 2021; Ainsworth and Rogers, 2007; Long et al., 2004). Multiple factors could be responsible for the reduced flux from water oxidation in PSII into the CBB carboxylation reaction: a decrease in the RuBisCO affinity for CO_2_, a decrease in CO_2_ delivery to RuBisCO in near-source plants, a lower RuBP availability/production rate, or a different availability/production rate of the cofactors required in the CBB cycle reactions, in particular ATP and NADPH. Considering our data, the most consistent explanation for the observed acclimation effects is upregulation of CEF relative to LEF as a response to the challenge of building up a proton gradient required for greater ATP production in an acidified high CO_2_ environment.

The effect on CEF regulation could not be verified in the field by P700^+^ absorption, owing to the low signal-to-noise ratio that prevented reliable measurements. In C3 flowering plants, carbonic anhydrases (CAs) catalyze the rapid, reversible interconversion of CO_2_ and HCO_3_^-^. They exist in three primary phylogenetic families α, β and γ, that functionally differ in terms of the kinetics of the forward/reverse reactions, and are localized in different compartments in leaves (Hines et al., 2021; Ignatova et al., 2019). Following CO_2_ entry into the leaf through stomata, these properties ensure smooth delivery by maintaining concentration gradients, rapidly shifting CO_2_ into soluble HCO_3_^-^ after traversing the cell membrane, and converting it back to CO_2_ precisely where the carbon-fixing enzymes of the Calvin cycle require it (Hines et al., 2021; Ignatova et al., 2019). Although we were unable to measure intracellular levels of these enzymes in the present study, adaptive changes of these enzymes in response to environmental CO_2_ levels should be considered as potential contributing factors to the effects observed herein.

**Figure 8:**
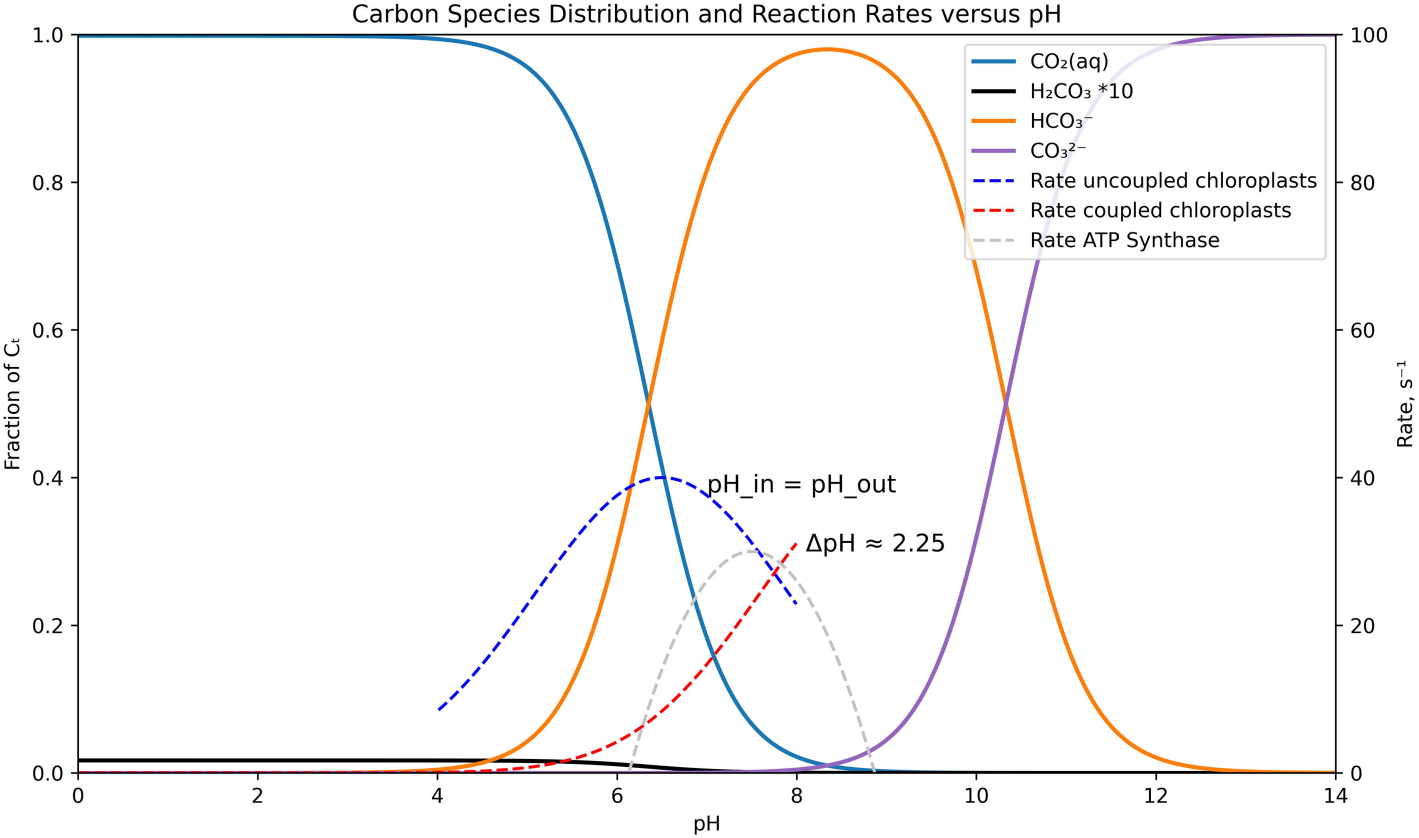
Chemical equilibrium curves (fractional speciation) for dissolved CO_2_ in water as a function of pH based on the reported equilibrium constants at 25 °C. The enzyme reaction rates for the intersystem electron transport rate through a plant-type Cytochrome b6f for uncoupled (dashed blue) and coupled (dashed red, ΔpH~2.25) chloroplasts, and the ATP synthesis rate through a chloroplast ATP-synthase (dashed grey) based on literature data (Tikhonov, 2014).

**Figure 8** shows the fractional concentrations of the four aqueous inorganic carbon species in equilibrium with water: CO_2_(aq), H_2_CO_3_(aq), HCO_3_^-^(aq), CO_3_^2-^(aq). Note that the CO_2_(aq), not carbonic acid (H_2_CO_3_), is the overwhelming source of aqueous acidification at pH values below ~6.5, and this equilibrates with CO_2_(mem) rapidly (Gutknecht et al., 1977). Hence, CO_2_-induced acidification is not just an aqueous pH decrease, but rather a multi-component acidification as defined in the Introduction. It collapses the pH gradient, owing to its fast transport across membranes (Gutknecht et al., 1977). CO_2_(aq) readily partitions into non-aqueous domains, where it can modify enzyme activity. Acting as a Lewis acid, it can dissolve in proteins and nucleic acids where it can carbamylate amino acids and nucleic acid bases (Bown, 1985; Ossola and Farmer, 2024). Upon increasing the partial pressure of CO_2_ in air from 400 ppm to 6,000 ppm, the concentration of CO_2_(aq) in pure water increases 15-fold from 1.3x10–^5^ M to 20x10–^5^ M, using the Henry’s Law equilibrium constant for the hydration reaction in water (Κ_H_ = 3.4×10–^2^ mol atm^-1^ at 25°C). The corresponding pH decreases by -0.9 units, from 5.93 to 5.02. Although the pH shift will be lower in the buffered cytoplasm of a leaf cell, the full effect of CO_2_ dissolution in cells must include the other dissolved forms of CO_2_ (Bown, 1985; Ossola and Farmer, 2024). Together, these multi-component acidification effects push enzymes outside of their optimum operating range. For example, the influence of pH alone on the intercellular electron transport rate through a plant-type Cytochrome b6f and the ATP synthesis rate through a chloroplast ATP-synthase as a function of intracellular pH is illustrated in **Figure 8** (Tikhonov, 2014). Both enzymes have a narrow optimum range (between ~pH 6–8 and pH 7.5-8.5, respectively), beyond which enzyme activity falls off rapidly.

## 5 Conclusions

In this study we aimed to answer the question how photosynthetic metabolism adapts upon exposure to extreme CO_2_ growth conditions near high CO_2_ emission sources in YNP. Our field measurements revealed significant increase in leaf thickness for plants growing in the high CO_2_ environment, but there were no changes in chlorophyll content measured in terms of SPAD. No change in Phi(II) was found, indicative of unaltered primary charge separation in PSII. Both Phi(NPQ) and LEF values were lower in plant leaves in the high CO_2_ environment, indicative of a shift in partitioning of light between photochemistry/heat and LEF/CEF rebalancing, respectively. pFv/Fm kinetics measured by a low light intensity FRRF protocol that probes bottlenecks in the PETC revealed that plants growing near the extreme CO_2_ sources are much slower to overcome bottlenecks in the PETC/CBB and reach lower pFv/Fm values and lower photosynthetic efficiency overall. We showed that these plants require higher CO_2_ levels to activate the CBB carboxylation reaction. We conclude that near-source plants acclimate metabolically by favoring CEF at ambient CO_2_ and switching to LEF only in high CO_2_. Second, near-source plants show more rapid restoration of PETC bottlenecks after a brief period of dark time (3–4 minutes). This tendency to return more rapidly to the dark-adapted state is indicative of a faster return to CEF. Electron transport through CEF retains electrons in the PQH_2_ pool instead of removing them through LEF into the NADP^+^ pool for utilization in the CBB carboxylation reaction. This adaptation to greater CEF at high CO_2_ growth conditions would serve as a coping mechanism to the multi-component acidification that compromises proton motive force generation. Upregulation of CEF is an adaptive response to enhance proton pumping to restore proton gradients and stimulate ATP production. This study examined the unique growth environments in Yellowstone NP to draw conclusions about adaptation to extreme CO_2_ levels in a natural growth environment. We found that extreme CO_2_ causes the CBB carboxylation reaction to desensitize, combined with effects of high CO_2_-induced multi-component acidification that stimulate proton pumping for ATP production.

## Data Availability

The datasets presented in this study can be found in online repositories. The names of the repository/repositories and accession number(s) can be found below: https://doi.org/10.4121/cd2dc766-3004-41a3-851c-0365f62c8578.

## References

[B1] Ainsworth E. A. Long S. P. (2021). 30 years of free-air carbon dioxide enrichment (FACE): What have we learned about future crop productivity and its potential for adaptation? [Review. Global Change Biol. 27, 27–49. doi: 10.1111/gcb.15375 33135850

[B2] Ainsworth E. A. Rogers A. (2007). The response of photosynthesis and stomatal conductance to rising [CO2]: Mechanisms and environmental interactions [Review. Plant Cell Environ. 30, 258–270. doi: 10.1111/j.1365-3040.2007.01641.x 17263773

[B3] Ananyev G. Dismukes G. C. . (2005). “ How fast can Photosystem II split water? Kinetic performance at high and low frequencies [Conference Paper,” in *Photosynthesis Research*. ( Springer Nature). vol. 84, 355–365. doi: 10.1007/s11120-004-7081-1 16049797

[B4] Ananyev G. Gates C. Dismukes G. C. (2018). The multiplicity of roles for (bi)carbonate in photosystem II operation in the hypercarbonate-requiring cyanobacterium Arthrospira maxima [Article. Photosynthetica 56, 217–228. doi: 10.1007/s11099-018-0781-0 30311153

[B5] Ananyev G. Veldhuis M.-C. Zournas A. Gates C. Dismukes G. C. (2026). An optimized fast repetition rate fluorometry method reveals photochemical flux bottlenecks in C3 photosynthesis that limit carboxylation and growth. Mol. Plant. doi: 10.1016/j.molp.2026.05.023 42231573

[B6] Bargar K. E. (1978). Geology and Thermal History of Mammoth Hot Springs, Yellowstone National Park, Wyoming ( USGS). Available online at: https://pubs.usgs.gov/bul/1444/report.pdf (Accessed December 23, 2025).

[B7] Belyaeva N. Bulychev A. Riznichenko G. Y. Rubin A. (2019). Analyzing both the fast and the slow phases of chlorophyll a fluorescence and P700 absorbance changes in dark-adapted and preilluminated pea leaves using a thylakoid membrane model. Photosynth. Res. 140, 1–19. doi: 10.1007/s11120-019-00627-8 30810971

[B8] Bergfeld D. Lowenstern J. B. Hunt A. G. Shanks W. C. P. Evans W. C. (2014). Usgs Scientific Investigations Report 2011–5012 Version 1.1. Menlo Park, C.A: USGS. Volcano Science Center, Yellowstone Volcano Observatory.

[B9] Blankenship R. E. Tiede D. Barber J. Brudvig G. W. Fleming G. Ghirardi M. . (2023). Comparing photosynthetic and photovoltaic efficiencies and recognizing the potential for improvement. Science. 332 (6031), 805–809. 10.1126/science.1200165 21566184

[B10] Bown A. W. (1985). CO2 and intracellular pH. Plant Cell Environ. 8, 459–465. doi: 10.1111/j.1365-3040.1985.tb01681.x 40046247

[B11] Dusenge M. E. Duarte A. G. Way D. A. (2019). Plant carbon metabolism and climate change: elevated CO2 and temperature impacts on photosynthesis, photorespiration and respiration. New Phytol. 221, 32–49. doi: 10.1111/nph.15283 29983005

[B12] Dusenge M. E. Madhavji S. Way D. A. (2020). Contrasting acclimation responses to elevated CO2 and warming between an evergreen and a deciduous boreal conifer [Article. Global Change Biol. 26, 3639–3657. doi: 10.1111/gcb.15084 32181545

[B13] Duysens L. N. M. Strehler H. E. (1963). “ Mechanism of two photochemical reactions in algae as studied by means of fluorescence,” in Studies on Microalgae and Photosynthetic Bacteria (Tokyo: University of Tokyo Press), 353–372.

[B14] Farquhar G. D. Von Caemmerer S. Berry J. A. (2001). Models of photosynthesis [Article. Plant Physiol. 125, 42–45. doi: 10.1104/pp.125.1.42 11154292 PMC1539321

[B15] Gorbunov M. Y. Kolber Z. S. Falkowski P. G. (1999). Measuring photosynthetic parameters in individual algal cells by Fast Repetition Rate fluorometry. Photosynth. Res. 62, 141–153. doi: 10.1023/a:1006360005033 41886696

[B16] Gorbunov M. Y. Kuzminov F. I. Fadeev V. V. Kim J. D. Falkowski P. G. (2011). A kinetic model of non-photochemical quenching in cyanobacteria. Biochim. Biophys. Acta (BBA)-Bioenergetics 1807, 1591–1599. doi: 10.1016/j.bbabio.2011.08.009 21907180

[B17] Gout E. Boisson A.-M. Aubert S. Douce R. Bligny R. (2001). Origin of the cytoplasmic pH changes during anaerobic stress in higher plant cells. Carbon-13 and phosphorous-31 nuclear magnetic resonance studies. Plant Physiol. 125, 912–925. doi: 10.1104/pp.125.2.912 11161048 PMC64892

[B18] Griffin K. L. Seemann J. R. (1996). Plants, CO2 and photosynthesis in the 21st century [Review. Chem. Biol. 3, 245–254. doi: 10.1016/S1074-5521(96)90104-0 8807852

[B19] Gutknecht J. Bisson M. A. Tosteson F. C. (1977). Diffusion of carbon dioxide through lipid bilayer membranes: effects of carbonic anhydrase, bicarbonate, and unstirred layers. J. Gen. Physiol. 69, 779–794. doi: 10.1085/jgp.69.6.779 408462 PMC2215341

[B20] Heber U. Wagner U. Kaiser W. Neimanis S. Bailey K. Walker D. (1994). Fast cytoplasmic pH regulation in acid-stressed leaves. Plant Cell Physiol. 35, 479–488. doi: 10.1093/oxfordjournals.pcp.a078619

[B21] Hines K. M. Chaudhari V. Edgeworth K. N. Owens T. G. Hanson M. R. (2021). Absence of carbonic anhydrase in chloroplasts affects C3 plant development but not photosynthesis. Proc. Natl. Acad. Sci. 118, e2107425118. doi: 10.1073/pnas.2107425118 34380739 PMC8379964

[B22] Hurwitz S. McCleskey R. B. Bergfeld D. Peek S. E. Susong D. D. Roth D. A. . (2020). Hydrothermal activity in the southwest yellowstone plateau volcanic field [Article. Geochem. Geophys. Geosyst. 21, e2019GC008848. doi: 10.1029/2019GC008848 29143083

[B23] Ignatova L. Rudenko N. Zhurikova E. Borisova-Mubarakshina M. Ivanov B. (2019). Carbonic anhydrases in photosynthesizing cells of C3 higher plants. Metabolites 9, 73. doi: 10.37247/pab.1.2020.4 30995746 PMC6523093

[B24] Kiirats O. Cruz J. A. Edwards G. E. Kramer D. M. (2009). Feedback limitation of photosynthesis at high CO2 acts by modulating the activity of the chloroplast ATP synthase. Funct. Plant Biol. 36, 893–901. doi: 10.1071/FP09129 32688700

[B25] Kramer D. M. Evans J. R. (2011). The importance of energy balance in improving photosynthetic productivity [Article. Plant Physiol. 155, 70–78. doi: 10.1104/pp.110.166652 21078862 PMC3075755

[B26] Kuhlgert S. Austic G. Zegarac R. Osei-Bonsu I. Hoh D. Chilvers M. I. . (2016). MultispeQ Beta: a tool for large-scale plant phenotyping connected to the open PhotosynQ network. R. Soc Open Sci. 3, 160592. doi: 10.1098/rsos.160592 27853580 PMC5099005

[B27] Leakey A. D. B. Ainsworth E. A. Bernacchi C. J. Rogers A. Long S. P. Ort D. R. (2009). Elevated CO2 effects on plant carbon, nitrogen, and water relations: Six important lessons from FACE [Conference Paper. J. Exp. Bot. 60, 2859–2876. doi: 10.1093/jxb/erp096 19401412

[B28] Long S. P. Ainsworth E. A. Rogers A. Ort D. R. (2004). Rising atmospheric carbon dioxide: plants FACE the future. Annu. Rev. Plant Biol. 55, 591–628. doi: 10.1146/annurev.arplant.55.031903.141610 15377233

[B29] Maschler J. Bialic-Murphy L. Wan J. Andresen L. C. Zohner C. M. Reich P. B. . (2022). Links across ecological scales: Plant biomass responses to elevated CO2 [Review. Global Change Biol. 28, 6115–6134. doi: 10.1111/gcb.16351 36069191 PMC9825951

[B30] Ossola R. Farmer D. (2024). The chemical landscape of leaf surfaces and its interaction with the atmosphere. Chem. Rev. 124, 5764–5794. doi: 10.1021/acs.chemrev.3c00763 38652704 PMC11082906

[B31] Prywes N. Phillips N. R. Tuck O. T. Valentin-Alvarado L. E. Savage D. F. (2023). Rubisco function, evolution, and engineering. Annu. Rev. Biochem. 92, 385–410. doi: 10.1146/annurev-biochem-040320-101244 37127263

[B32] Qian X. Liu L. Croft H. Chen J. (2021). Relationship between leaf maximum carboxylation rate and chlorophyll content preserved across 13 species [Article. J. Geophysical Research: Biogeosciences 126, e2020JG006076. doi: 10.1029/2020JG006076 29143083

[B33] Raines C. A. Cavanagh A. P. Simkin A. J. (2022). “ Improving carbon fixation,” in Photosynthesis in Action (London: Elsevier), 175–192.

[B34] Schansker G. Tóth S. Z. Holzwarth A. R. Garab G. (2014). Chlorophyll a fluorescence: beyond the limits of the QA model. Photosynth. Res. 120, 43–58. doi: 10.1007/s11120-013-9806-5 23456268

[B35] Schreiber U. Bilger W. Neubauer C. (1995). “ Chlorophyll fluorescence as a nonintrusive indicator for rapid assessment of *in vivo* photosynthesis,” in Ecophysiology of Photosynthesis. Eds. Schulze E.-D. Caldwell M. M. (Berlin: Springer Berlin Heidelberg), 49–70. doi: 10.1007/978-3-642-79354-7_3

[B36] Sharkey T. D. (2024). The end game(s) of photosynthetic carbon metabolism. Plant Physiol. 195, 67–78. doi: 10.1093/plphys/kiad601 38163636 PMC11060661

[B37] Sharkey T. D. Berry J. A. Sage R. F. (1988). Regulation of photosynthetic electron-transport in Phaseolus vulgaris L. as determined by room-temperature chlorophyll a fluorescence [Article. Planta 176, 415–424. doi: 10.1007/BF00395423 24220871

[B38] Sims D. A. Luo Y. Seemann J. R. (1998). Importance of leaf versus whole plant CO2 environment for photosynthetic acclimation. Plant Cell Environ. 21, 1189–1196. doi: 10.1046/j.1365-3040.1998.00377.x 37945311

[B39] Suzuki Y. Ishiyama K. Sugawara M. Suzuki Y. Kondo E. Takegahara-Tamakawa Y. . (2020). Overproduction of chloroplast glyceraldehyde-3-phosphate dehydrogenase improves photosynthesis slightly under elevated [CO2] conditions in rice. Plant Cell Physiol. 62, 156–165. doi: 10.1093/pcp/pcaa149 33289530

[B40] Sweetlove L. J. George Ratcliffe R. (2011). Flux-balance modeling of plant metabolism [Article. Front. Plant Sci. 2, 38. doi: 10.3389/fpls.2011.00038 22645533 PMC3355794

[B41] Tikhonov A. N. (2014). The cytochrome b6f complex at the crossroad of photosynthetic electron transport pathways. Plant Physiol. Biochem. 81, 163–183. doi: 10.1016/j.plaphy.2013.12.011 24485217

[B42] Wagner U. Kolbowski J. Oja V. Laisk A. Heber U. (1990). pH homeostasis of the chloroplast stroma can protect photosynthesis of leaves during the influx of potentially acidic gases. Biochim. Biophys. Acta (BBA)-Bioenergetics 1016, 115–120. doi: 10.1016/0005-2728(90)90013-t

[B43] Werner C. Brantley S. (2003). CO2 emissions from the Yellowstone volcanic system [Article. Geochem. Geophys. Geosyst. 4, 1061. doi: 10.1029/2002CG000473 29143083

[B44] Wu A. Brider J. Busch F. A. Chen M. Chenu K. Clarke V. C. . (2023). A cross-scale analysis to understand and quantify the effects of photosynthetic enhancement on crop growth and yield across environments [Article. Plant Cell Environ. 46, 23–44. doi: 10.1111/pce.14453 36200623 PMC10091820

[B45] Young-Mathews A. (2012). Plant Fact Sheet for Lance Selfheal (Prunella Vulgaris Ssp. Lanceolata). Corvalis, OR: USDA Natural Resources Conservation Service, Corvallis Plant Materials Center.

